# Linking Free Text Documentation of Functioning and Disability to the ICF With Natural Language Processing

**DOI:** 10.3389/fresc.2021.742702

**Published:** 2021-11-05

**Authors:** Denis Newman-Griffis, Jonathan Camacho Maldonado, Pei-Shu Ho, Maryanne Sacco, Rafael Jimenez Silva, Julia Porcino, Leighton Chan

**Affiliations:** ^1^Rehabilitation Medicine Department, National Institutes of Health Clinical Center, Bethesda, MD, United States; ^2^Department of Biomedical Informatics, University of Pittsburgh, Pittsburgh, PA, United States

**Keywords:** natural language processing, clinical coding, disability evaluation, international classification of functioning disability and health, electronic health records, artificial intelligence, functional status, ICF

## Abstract

**Background:** Invaluable information on patient functioning and the complex interactions that define it is recorded in free text portions of the Electronic Health Record (EHR). Leveraging this information to improve clinical decision-making and conduct research requires natural language processing (NLP) technologies to identify and organize the information recorded in clinical documentation.

**Methods:** We used natural language processing methods to analyze information about patient functioning recorded in two collections of clinical documents pertaining to claims for federal disability benefits from the U.S. Social Security Administration (SSA). We grounded our analysis in the International Classification of Functioning, Disability, and Health (ICF), and used the Activities and Participation domain of the ICF to classify information about functioning in three key areas: mobility, self-care, and domestic life. After annotating functional status information in our datasets through expert clinical review, we trained machine learning-based NLP models to automatically assign ICF categories to mentions of functional activity.

**Results:** We found that rich and diverse information on patient functioning was documented in the free text records. Annotation of 289 documents for Mobility information yielded 2,455 mentions of Mobility activities and 3,176 specific actions corresponding to 13 ICF-based categories. Annotation of 329 documents for Self-Care and Domestic Life information yielded 3,990 activity mentions and 4,665 specific actions corresponding to 16 ICF-based categories. NLP systems for automated ICF coding achieved over 80% macro-averaged F-measure on both datasets, indicating strong performance across all ICF categories used.

**Conclusions:** Natural language processing can help to navigate the tradeoff between flexible and expressive clinical documentation of functioning and standardizable data for comparability and learning. The ICF has practical limitations for classifying functional status information in clinical documentation but presents a valuable framework for organizing the information recorded in health records about patient functioning. This study advances the development of robust, ICF-based NLP technologies to analyze information on patient functioning and has significant implications for NLP-powered analysis of functional status information in disability benefits management, clinical care, and research.

## Introduction

A person's functioning requires a multifaceted picture of the complex interactions between the person and the world around them. The International Classification of Functioning, Disability and Health (ICF) (1) conceptualizes these interactions as between health condition(s), body structures and functions, activities and participation, and both environmental and personal contextual factors of a person. In order to fully capture the multifactorial nature of functional outcomes and a person's experience of their functioning, providers primarily turn to free text documentation in the Electronic Health Record (EHR) (2–4). While the flexibility of free text presents a barrier to standardization in the EHR, limiting comparability across patients and opportunities for data-driven learning in modern health systems (5), the expressivity of natural language is the key to capturing the nuances of functioning as it is experienced in the life of the patient (6). For example, two patients reporting moderate limitations in walking may experience them in entirely different ways: One may describe arthritic stiffness in their knees that causes manageable discomfort in navigating employment in an office, while chronic low back pain of another patient makes their hiking hobby no longer viable. These differences in experience, which inform both therapeutic interventions and the perception of the patient of their own functioning, are difficult to capture in standardized instruments but can be easily described in natural language.

How to navigate the tradeoff between flexibility in clinical documentation and standardization for comparability and learning? We explored the use of natural language processing (NLP) systems, grounded in the ICF, to index and organize information about functioning and disability in free text clinical records, enabling a measure of standardization without sacrificing the details of the patient experience. NLP can be used to identify, organize, and retrieve information from free text documents for use in clinical decision-making and research (7, 8). NLP shows growing promise for capturing and analyzing information on functioning: Kukafka et al. (9) developed an early system for coding rehabilitation discharge summaries to identify activities including eating, dressing, and toileting, and NLP has since been used for a variety of purposes, including locating functional status documentation in oncology notes (10), identifying potential wheelchair use (11), and detecting functional outcomes of geriatric syndrome (12). We have previously developed NLP methods to identify activity mentions describing mobility functioning in clinical notes (13–15) and to link these activity mentions to the Mobility chapter of the Activities and Participation domain of the ICF (16).

This study investigated NLP methods for automatically coding documentation of key domains of functioning to the ICF and evaluated their performance on coding medical records associated with claims for federal disability benefits submitted to the U.S. Social Security Administration (SSA). We adapted our previous work on Mobility information to expand to information from the Self-Care and Domestic Life chapters of the Activities and Participation domain of the ICF. Together with Mobility, these domains align with the majority of Activities of Daily Living (ADLs)—fundamental activities frequently considered in therapeutic patient assessment, such as dressing, hygiene, eating, and ambulation—(17, 18), and account for 11 of the 18 items in the Functional Independence Measure (FIM)—a tool for assessing the degree of independence of a patient, commonly used in assessing rehabilitation outcomes (19). Thus, NLP methods to automatically identify activities in these three ICF chapters have significant potential for use in clinical information systems.

The remainder of this article is organized as follows: In the Materials and Methods section, we describe the medical records we analyzed from SSA disability benefits claims and present the NLP methods used for linking information about patient function in these records to relevant categories in the ICF. The Results section presents our experimental findings and analysis of successes and challenges in coding clinical data with the ICF. The Discussion section outlines implications from our work, including challenges for applying the ICF in coding clinical notes, opportunities for NLP impact in the SSA disability adjudication process and in broader clinical information systems, and limitations of the study.

## Materials and Methods

Our study involved the development and evaluation of machine learning-based statistical models for linking descriptions of Mobility, Self-Care, and Domestic Life functioning in free text clinical documentation to relevant categories in the ICF. While we considered an automated assignment of the qualifier component of ICF codes out of scope for this study and used two-level classification categories for the output of our NLP systems, we referred to this process as *ICF coding* to align it with prior literature on automated medical coding systems. We used the term *functional status information* (FSI) to refer to information about patient functioning, including specific observations in activity mentions.

### Data Sources and Use of the ICF

Our primary data source for this study was free text medical records collected by SSA in the process of adjudicating federal disability benefits claims. During the adjudication process of a claim of an individual, SSA may obtain records from prior medical encounters of that individual in order to collect medical evidence related to the disability claim. These records are reviewed by expert adjudicators at SSA to identify appropriate evidence to support the claim decision, such as impairment history and severity, relationship to work requirements, etc. The volume of these records is substantial, with each claim having potentially hundreds or thousands of pages of associated medical records, presenting a significant opportunity for NLP methods to assist in evidence review by automatically identifying relevant information.

We used two types of medical documents in the study. (1) Consultative Examination (CE) reports are written by a medical expert commissioned by SSA to examine a claimant in-depth as part of the claim adjudication process. (2) EHR data are provided directly to SSA by health providers pursuant to a disability benefits claim. Both types of documents are frequently submitted to SSA as faxed or scanned documents and thus require Optical Character Recognition (OCR) to convert them to text for NLP analysis. All documents used in this study were converted to text using the Nuance OmniPage™ (now Kofax OmniPage Ultimate™) OCR software.

We selected the ICF, and the Activities and Participation domain in particular, as our framework for identifying functioning information in these documents. We chose the ICF due to its role as an internationally recognized coding system for functioning, and our familiarity with it (6, 15, 16). SSA assesses function as part of the claim adjudication process, including assessment of residual functional capacity for individuals applying for disability benefits, examining both physical and mental function. We identified the Mobility, Self-Care, and Domestic Life chapters of the ICF as being most relevant to this process and the types of functioning documented most frequently in the data we reviewed. As noted in the Introduction, these chapters are also closely aligned with commonly used ADL measures and the FIM, making them particularly relevant types of information to study for a broad range of information needs in rehabilitation. We used the title case in this article to refer to Mobility, Self-Care, and Domestic Life information, as defined by the ICF, to distinguish from the more general uses of these terms.

#### Document Collections for Annotation

We identified two sets of medical documents from SSA to annotate for Mobility, Self-Care, and Domestic Life FSI. Both datasets for annotation were drawn from adult disability benefits claims with a decision issued in 2016–2018, primarily related to musculoskeletal, neurological, or mental impairments.

Following our prior work on analyzing Mobility information (15), we identified 300 CEs likely to contain descriptions of Mobility functioning. We ensured that each CE corresponded to a different claimant in order to control for cross-document correlation from an individual claimant.

An additional 350 documents were then selected to annotate for Self-Care and Domestic Life information. The documents were selected from the same overall set of claims as the Mobility documents, but we ensured that the specific claims used in annotation were disjoint between the two datasets. As the concepts of Self-Care and Domestic Life are highly intertwined and often discussed together in clinical notes—e.g., eating (Self-Care) and preparing meals and cleaning (Domestic Life)—we chose to annotate for these chapters jointly (referred to in the remainder of the article as “Self-Care/Domestic Life”). Annotated documents included both CEs and EHR data; no two documents of the same type were included for any individual claimant.

#### SSA Document Collection for Computational Language Modeling

A further set of 65,514 documents collected by SSA was used for machine learning of statistical models of clinical language as used in the SSA setting (as detailed in the “Text representation with word embeddings” section below). Many documents included in this collection included notes from multiple clinical encounters during the history of a patient with a particular healthcare provider. Each “document” was thus much longer on average than a single clinical note, with a median document length of 3,476 words. These documents were sampled by SSA separately from the documents used for annotation, using a broader set of criteria to enhance the diversity of the data: adult claims adjudicated based on musculoskeletal, neurological, or mental impairments, with a decision issued during 2013–2018, drawn from multiple states around the U.S. We confirmed that no documents selected for Mobility or Self-Care/Domestic Life annotations were included in this collection.

### Annotation Process

**Figure 1:**
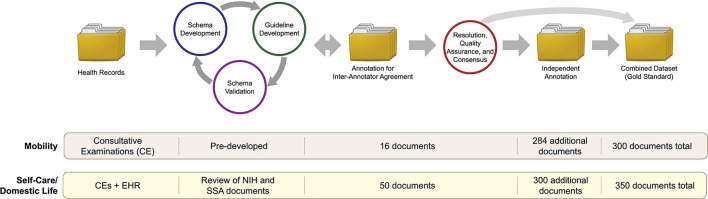
Flowchart illustration of the annotation process. Data sources and document counts are provided for Mobility and Self-Care/Domestic Life annotations separately.

Annotation of SSA documents for FSI regarding Mobility and Self-Care/Domestic Life was performed in a multistage process, illustrated in Figure 1. Mobility information was annotated using guidelines developed in previous work (15); we adapted this existing process to develop new guidelines for Self-Care/Domestic Life information. We developed the annotation guidelines *via* an iterative process among the annotators (JCM, PSH, MS, and RJS), involving team annotation and discussion to refine a schema for representing Self-Care/Domestic Life information and develop clear guidelines for how to annotate for it in free text. After guideline development, the annotators jointly annotated a small set of documents (50 for the new Self-Care/Domestic Life guidelines, and 16 to further validate the existing Mobility guidelines in SSA data), and Inter-Annotator Agreement (IAA) was calculated (IAA values are reported with other dataset statistics in the Results section). Following standard practice in annotating for text spans (20, 21), we calculated IAA using the F-1 measure. Disagreements were then resolved by joint meetings among the annotators to produce a final consensus version of the jointly annotated documents. Finally, each individual annotator annotated a further set of documents independently, which were then combined with the consensus annotations to produce the final “gold standard” annotated corpus.

When annotating a document, the first step in our process was to identify *activity mentions*, which we operationalized as self-contained spans of text describing the functioning person's within the scope of the relevant ICF Activities and Participation chapters. Within each activity mention, we then identified each distinct *action* referred to, operationalized as a distinct activity defined by one of the ICF categories within the relevant chapters of the two-level ICF classification (or activity of similar granularity not specifically captured in the ICF, e.g., “do household chores”). These categories are represented using the ICF format of the letter *d* (indicating the Activities and Participation domain), followed by three digits: a one-digit chapter identifier and a two-digit category identifier (e.g., d450 indicates the *Walking* category in Chapter 4 *Mobility*). We referred to these as *second-level categories* to distinguish them from the more specific subcategories in the detailed classification (e.g., d4501 *Walking long distances*).

**Figure 2:**
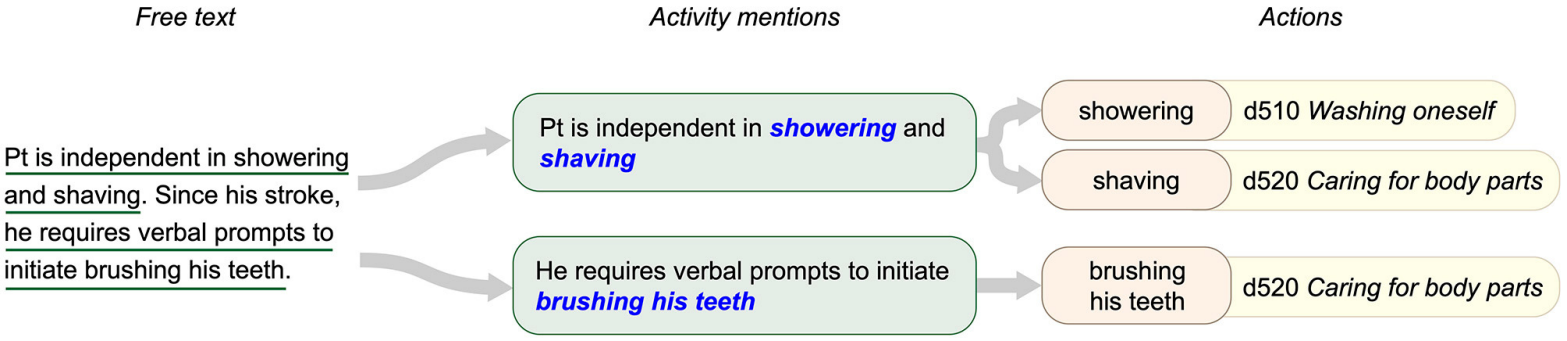
Structure of annotations for functional status information. Free text is annotated to identify activity mentions describing specific observations. Each activity mention may include one or more Action components, which can be mapped to second-level ICF categories.

Each of the identified action components (which we denote with a capitalized *Action* for the remainder of this article, for clarity) within an activity mention was then assigned the second-level ICF category best representing the activity described. We excluded the “other specified” and “unspecified” ICF categories, such as d598 *Self-care, other specified* and d599 *Self-care, other unspecified*, from use in annotation due to their ambiguity. In cases where an Action component referred to an activity for which no specific ICF category was appropriate (e.g., “doing household tasks”), or when multiple categories could apply (e.g., “denies difficulty with ADLs”), a label of “Other” was used. Figure 2 provides an illustrated example of Self-Care/Domestic Life activity mentions, including one with two Action components.

The focus of annotation was on observations or descriptions of specific, volitional activities performed by the patient within the specific domains of interest. We, therefore, excluded the following types of information about functioning: (1) hypothetical statements (e.g., “her sleep is better if she takes medication”); (2) education given by the provider (e.g., “The patient educated on how he can attempt to dress his lower body in bed”); and (3) references to habitual activity in the context of work duties (e.g., “his job at the hotel involves doing laundry and cleaning guest rooms”).

#### Patient Engagement in Medication Management and Non-Pharmacological Therapies as Categories of Self-Care

The documents reviewed for Self-Care/Domestic Life guideline development included frequent discussions of active engagement of patients in the therapeutic process, including adherence to medication management regimens and participation in non-pharmacological therapies. While these mentions provided valuable evidence of distinct kinds of patient engagement in self-care, they were not reflected by ICF categories more specific than d570 *Looking after one's health*. To more accurately capture—and differentiate between—these frequent topics, we added two additional Action labels based on codes in the Systematized Nomenclature of Medicine Clinical Terms set (SNOMED CT). We used *Manage medication* (SNOMED CT code 285033005) to refer to anything related to compliance with medications such as the ability to store medications, obtain medications, take the medications, etc. This label also included the mismanagement of medication (e.g., forgetting to take prescribed medications). We used *Therapy* (SNOMED CT code 709007004) to refer to attending or, otherwise, engaging in non-pharmacological therapies, such as addiction treatment programs, physical therapy, occupational therapy, cognitive behavioral therapy, psychological therapy, and anger management. We did not use these labels to annotate the therapeutic interventions themselves, which are out of the scope of the ICF. Thus, while a mention of a patient attending physical therapy was annotated as a *Therapy* activity mention, a mention of a physical therapy appointment with no indication of whether the patient attended or not did not provide evidence of self-care and was not annotated.

### Methods for Automated ICF Coding

**Figure 3:**
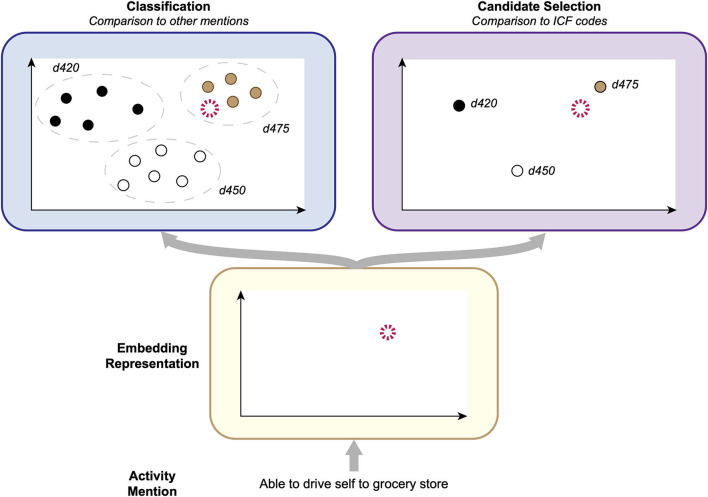
Conceptual illustration of the ICF coding process. Given an activity mention, an embedding representation of the report is calculated and then compared with other activity mentions, in Classification; or available ICF categories, in Candidate Selection.

We experimented with two strategies to develop computer methods to automatically assign ICF categories to Mobility and Self-Care/Domestic Life activity mentions. In our prior work (16), we explored a variety of methods for ICF coding, including both *classification*—identifying the group of samples a given activity mention is most similar to—and *candidate selection*—identifying which ICF category a given activity mention is most similar to—approaches, for Mobility information only. In this study, we evaluated the best-performing classification and candidate selection models from this prior work on the SSA datasets we developed for Mobility and Self-Care/Domestic Life. Our overall process is illustrated in Figure 3.

#### Text Representation With Word Embeddings

Given an activity mention, we calculated a numeric representation of the text using word embedding features. In word embedding models, each word and phrase is represented mathematically using a vector of *n* real numbers—frequent values for *n* include 100, 300, and 768—with the property that words that are similar in meaning generally have similar numeric representations (22). These models are fundamental resources for modern NLP methods. Our prior work demonstrated that word embedding features alone were more informative for ICF coding than features indicating the presence and/or frequency of specific words (referred to as *lexical features*) or combined embedding and lexical features (16); we, therefore, used word embedding features alone in this study. We experimented with two methods for word embedding:

- In *static* embeddings, each unique word is represented by a single vector. Thus, for example, every occurrence of the word *patient* is represented within the model using the same set of real numbers. We used FastText (23), a commonly used method that integrates sub-word information into embedding learning to better capture morphological patterns.
- In *contextualized* embeddings, each word is represented by a single vector conditioned on the context it appears in; thus, the word “cold” in “patient described cold symptoms” and “applied a cold pack” is represented using different vectors of real numbers for each case. This provides additional context sensitivity in how the model represents text content. We used BERT (24), a recent embedding model that has rapidly become the *de facto* standard for text representation in NLP.

The parameters of both static and contextualized embedding models (i.e., the values used to represent words and phrases) are typically estimated prior to their usage in any specific NLP task (e.g., our ICF coding application), based on a large sample of natural language (referred to as a *corpus*). Different corpora may be chosen for different purposes—e.g., estimating an embedding model using the text of PubMed abstracts provides useful representations for analyzing scientific literature while using the text of clinical notes provides more useful representations for clinical applications. We, therefore, experimented with multiple corpora to estimate our word embedding models (referred to in machine learning as *model training*); each of which reflects different tradeoffs between corpus size and representativeness for the target task. These corpora are summarized in Table 1.

**Table 1:** Free text corpora used to train word embedding models for text representation.

**Training corpus**	**Number of notes**	**Number of words (approx.)**	**Data description**
MIMIC	2,083,180	497 million	Critical care admissions (26). Most commonly used corpus for language modeling in clinical NLP.
NIHCC	63,605	11.8 million	Physical therapy and occupational therapy encounters, used in our prior work on coding Mobility information to the ICF (16).
SSA	65,514	664 million	Clinical data associated with disability benefits claims submitted to SSA. New in this study.

*MIMIC-III was used to train both FastText and BERT models; NIHCC and SSA were used for FastText embeddings only*.

For static word embeddings, we experimented with three clinical corpora for training embedding models. In each case, document texts were broken down into individual words (tokenized) with the spaCy software (25), and the following processing steps were applied to normalize out aspects of the text irrelevant to our language modeling goal: all words were converted to lowercase, all numbers were normalized to “[NUMBER]”, all URLs were normalized to “[URL],” and all dates and times were normalized to “[DATE]” and “[TIME],” respectively. The FastText software (version 0.2.0) was used with the skipgram algorithm, 300-dimensional embeddings, and all other settings at default to training embeddings on the following three corpora:

- **MIMIC**: Approximately 2 million free text notes are included in the publicly available Medical Information Mart for Intensive Care (MIMIC) critical care database, version 3 (26). Notes are associated with admissions to ICU units of Beth Israel Deaconess Medical Center in Boston between 2001 and 2012 and are commonly used for language modeling in clinical NLP research.
- **NIHCC**: Over 63,000 free text notes from 10 years of Physical Therapy and Occupational Therapy encounters in the Rehabilitation Medicine Department of the NIH Clinical Center were collected and used for calculating word embedding features in our previous work (16).
- **SSA**: Over 65,000 free text notes associated with disability claims processed by SSA within a 5-year period (as described in the “SSA document collection for the language modeling” section above).

Contextualized embedding models require significant computing power to train on new data, and pre-trained models are typically used to generate text features. We used the clinicalBERT model released by Alsentzer et al. (27), which was trained on MIMIC clinical notes and produces 768-dimensional word embeddings.

##### Action Oracle

As illustrated in Figure 2, activity mentions are complex statements, including multiple pieces of information. Thieu et al. (15) define sub-components of activity mentions, including (1) a source of Assistance—typically a device, person, or structure in the physical environment used in activity performance; (2) a Quantification—an objective measure of functional performance, such as distance or time; and (3) one or more specific Actions being performed, which correspond to defined activities in the ICF Activities and Participation domain. For example, the activity mention “Pt ambulated 300' in a clinic with a rolling walker” which includes the Action component “ambulated,” the Assistance component “with a rolling walker,” and the Quantification component “300'.” Action components are annotated with the second-level ICF categories, which the NLP systems described in this study are designed to assign.

Prior work on extracting activity mentions from the free text (13, 14) did not include extraction of the Action sub-components. However, as NLP methods for functional status information continue to develop, more complex models that reflect the semantic structure of activity mentions will be needed. We, therefore, evaluated the ICF coding models in this study in two settings: (1) an *Action oracle* setting, in which both an activity mention and the location of an Action component within it (i.e., where, in the text span of the activity mention, the Action is found) are input to the ICF coding model; and (2) a non-oracle setting in which only the activity mention is provided (reflecting the technologies so far developed for extracting activity mentions).

#### Classification

In classification approaches, a mathematical representation is calculated for each activity mention using word-embedding features, and a predictive model is trained to assign an ICF category to each Action component based on its similarity to previously observed samples labeled with each ICF category. We adopted the best-performing classification model from our prior work (16), a Support Vector Machine (28) using a word embedding features as input. Given an input activity mention, we calculated its embedding features in one of four ways:

- **Static embeddings, no Action oracle**: the activity mention is represented by averaging the word embeddings of each word in the mention.
- **Static embeddings, with Action oracle**: two averaged embeddings are calculated: (1) the averaged embedding for the words in the Action component; and (2) the average of other all words in the activity mention. These are concatenated, i.e., combined into a single, longer vector, to produce the final representation.
- **Contextualized embeddings, no Action oracle**: the activity mention is represented as the averaged context-sensitive embeddings for each of its words.
- **Contextualized embeddings, with Action oracle**: as the contextualized embeddings of words in the Action component already reflect information about the full activity mention, we averaged the embeddings of Action component words only.

#### Candidate Selection

In the candidate selection approach, an embedding representation is calculated for each activity mention and is then compared to embedding representations of each of the available ICF categories to identify which category the given mention is most similar to. We adopted the best-performing candidate selection model from our prior work (16), consisting of a Deep Neural Network (DNN) that operates as follows:

1. The model takes as input an activity mention embedding and embedding representations of the ICF categories that could be assigned to it (i.e., all Mobility categories or all Self-Care/Domestic Life categories).
2. These embeddings are all fed into a DNN to calculate new embedding representations of the candidate ICF categories, conditioned on this specific activity mention.
3. The conditional ICF category embeddings are compared with the activity mention embedding using the cosine similarity measure, and the category with the highest similarity is chosen as the model output.

Embedding features of activity mentions were calculated using the strategies described in the “Classification” section. Embedding representations of ICF categories were calculated as the averaged embeddings of each word in the definition of the category presented in the ICF, using both static and contextualized embeddings. For the “Other” label, the following definitions were used: “Mobility other or unspecified” for Mobility, and “Self-care or domestic life other or unspecified” for Self-Care/Domestic Life. For the added *Therapy* and *Manage medication* labels, we used the names of the corresponding SNOMED CT codes (“Ability to manage medication” and “Compliance behavior to the therapeutic regimen,” respectively). Further details of the model are presented in (16). Following our prior work, we used a 3-layer DNN with hidden layer size 300 when using static embedding features without the Action oracle, a 3-layer DNN with layer size 600 when using static embeddings with the Action oracle (to match the dimensionality of the concatenated activity mention and Action component embeddings), and a 1-layer DNN with layer size 768 when using BERT embedding features (for which vector dimensionality does not change with the Action oracle).

### Experimental Procedure

Prior to machine learning experiments, each dataset was split at the document level into training data, for training the machine learning models, and test data for evaluating them. Test documents were sampled to include at least 20% of the samples for each ICF category. Statistical significance testing was performed using the bootstrap resampling method with 1,000 replicates, which is commonly used to analyze performance metrics in NLP research (29, 30).

#### Development Experiments

Training data were further split into 10-fold for development experiments to select the best word embedding method for classification and candidate selection approaches. For development experiments, cross validation was used; models were trained on 9-fold (90% of the training data) and evaluated on the held-out 10th fold, and this process was then repeated to evaluate on each of the 10-fold, with model performance being averaged across the folds to calculate final values. Model performance was calculated using the F-1 score (20), calculated as the harmonic mean between precision (positive predictive value) and recall (sensitivity). F-1 score was calculated for each ICF category in each dataset and averaged across categories to calculate macro F-1. The embeddings producing the highest macro F-1 on the development experiments were chosen to use for the main experiments.

#### Main Experiments and Model Evaluation

Once final word embeddings were chosen, an additional classification and candidate selection model was trained for each of the Mobility and Self-Care/Domestic Life datasets, using all of the training data. These models were then evaluated on the held-out test documents, with performance measured using F-1 for each individual ICF category, and overall performance calculated as macro-averaged F-1 score.

## Results

### Annotated Datasets

Table 2 presents the overall statistics of the two SSA datasets annotated for functional status information. Several of the documents selected for annotation were omitted after conversion to text with the OCR software due to failures in the OCR conversion, resulting in a total of 289 documents annotated for Mobility, and 329 documents annotated for Self-Care/Domestic Life. The majority of documents were found to contain descriptions of the target types of functioning: 251/289 (87%) of Mobility documents and 285/329 (87%) of Self-Care/Domestic Life documents contained at least one activity mention pertaining to the relevant ICF chapters. Each activity mention could contain zero, one, or more than one Action component; a total of 3,176 Actions were annotated for Mobility and 4,665 for Self-Care/Domestic Life. Only 132 Mobility activity mentions (5.4% of the total) and 134 Self-Care/Domestic Life activity mentions (3.4% of the total) were found to not contain any specific Action components. Inter-annotator agreement (IAA) was found to be 0.778 F-1 for Mobility and 0.695 F-1 for Self-Care/Domestic Life, comparable to IAA calculated in our previous study on annotating Mobility information in clinical reports (15). ICF coding annotation has previously been found to yield high agreement for resources and goals as well as specific problems (31). The two datasets are described in greater detail in the following sections.

**Table 2:** Datasets of documents annotated for functional status information, drawn from U.S. Social Security Administration disability benefits cases.

	**Mobility**	**Self-Care/Domestic Life**
Number of documents annotated	289	329
With activity mentions	251	285
Total activity mentions	2,455	3,990
Including at least one Action	2,323 (94.6%)	3,866 (96.9%)
Total number of Actions	3,176	4,665
Training set size (documents / Actions)	203 / 2,361	229 / 3,350
Test set size (documents / Actions)	45 / 815	56 / 1,315

*Separate sets of documents were annotated for Mobility (ICF Activities and Participation Chapter 4) and Self-Care/Domestic Life (ICF Activities and Participation Chapters 5 and 6)*.

#### Mobility Dataset

A total of 12 unique second-level ICF categories were used for annotating Mobility information; Table 3 lists the frequency of each of these categories in the annotated dataset, together with the “Other” category. Of the categories in the Mobility chapter, only d480 *Riding animals for transportation* was not observed in the annotation process. d465 *Moving around using equipment* was excluded from annotation, as the use of equipment was annotated using Assistance components of Mobility activity mentions; d455 *Moving around* was used instead. The most frequent categories were d450 *Walking* (23% of Actions), d410 *Changing basic body position* (17.6% of Actions), and d415 *Maintaining a body position* (16% of Actions). Only d420 *Transferring oneself*, d435 *Moving objects with lower extremities*, and d460 *Moving around in different locations* were observed fewer than 100 times. A total of 123 samples (3.9% of Actions) were found that could not be mapped to a single appropriate second-level ICF category. These included Actions, which could map to multiple categories, such as “The patient is able to ambulate in the hallway and stairs” (which can refer to both d450 *Walking* and d460 *Moving around in different locations*), and Actions, which were too vague to map to any specific categories, such as “The patient cannot manage/negotiate stairs.”

**Table 3:** ICF category descriptions and frequencies for Mobility dataset (3,176 samples total).

**Mobility category**	**Description**	**Frequency**	**% of all samples**	**Training samples**	**Test samples**
d450	Walking	730	23.0%	559 (77%)	171 (23%)
d410	Changing basic body position	560	17.6%	419 (75%)	141 (25%)
d415	Maintaining a body position	508	16.0%	385 (76%)	123 (24%)
d440	Fine hand use	319	10.0%	247 (77%)	72 (23%)
d430	Lifting and carrying objects	244	7.7%	167 (68%)	77 (32%)
d475	Driving	215	6.8%	165 (77%)	50 (23%)
d445	Hand and arm use	163	5.1%	104 (64%)	59 (36%)
d455	Moving around	147	4.6%	99 (67%)	48 (33%)
Other	Mobility-related activities for which no specific ICF category could be identified	123	3.9%	96 (78%)	27 (22%)
d470	Using transportation	103	3.2%	80 (78%)	23 (22%)
d460	Moving around in different locations	55	1.7%	34 (62%)	21 (38%)
d435	Moving objects with lower extremities	5	0.2%	4 (80%)	1 (20%)
d420	Transferring oneself	4	0.2%	2 (50%)	2 (50%)

*Categories are ordered by frequency in the dataset. Sample count and relative distribution between training data (203 documents, 2,361 samples) and test data (45 documents, 815 samples) are given for each category. Descriptions given are the preferred name of each category in the ICF*.

#### Self-Care/Domestic Life Dataset

Thirteen distinct second-level ICF categories (seven from Chapter 5 *Self-Care*, six from Chapter 6 *Domestic Life*) were used in data annotation, together with the added labels of *Manage medication* and *Therapy* and the “Other” category. Table 4 lists the observed frequency of each of these labels in the dataset. The most frequent category was d570 *Looking after one's health*, accounting for 43.6% of the samples by itself. Five categories (d530 *Toileting*, d560 *Drinking*, d610 *Acquiring a place to live*, d650 *Caring for household objects*, and d660 *Assisting others*) occurred fewer than 100 times. A total of 175 samples were found that could not be mapped to a single appropriate second-level ICF category, such as “The patient is independent with ADLs” (which includes multiple Self-Care activities).

**Table 4:** ICF category descriptions and frequencies for Self-Care/Domestic Life dataset (4,665 samples total).

**Self-care/domestic life category**	**Description**	**Frequency**	**% of all samples**	**Training samples**	**Test samples**
d570	Looking after one's health	2,032	43.6%	1,496 (74%)	536 (26%)
Manage medication	Ability to manage medication (SNOMED CT code 285033005)	520	11.1%	359 (69%)	161 (31%)
d540	Dressing	353	7.6%	268 (76%)	85 (24%)
d520	Caring for body parts	312	6.7%	228 (73%)	84 (27%)
d640	Doing housework	297	6.4%	205 (69%)	92 (31%)
d630	Preparing meals	222	4.8%	165 (74%)	57 (26%)
Other	Self-Care/Domestic Life activities for which no specific ICF category could be identified	174	3.7%	127 (73%)	47 (27%)
Therapy	Compliance behavior to therapeutic regimen (SNOMED CT code 709007004)	143	3.1%	103 (72%)	40 (28%)
d620	Acquisition of goods and services	142	3.0%	101 (71%)	41 (29%)
d510	Washing oneself	121	2.6%	90 (74%)	31 (26%)
d550	Eating	102	2.2%	57 (56%)	45 (44%)
d560	Drinking	82	1.8%	60 (73%)	22 (27%)
d660	Assisting others	79	1.7%	46 (58%)	33 (42%)
d650	Caring for household objects	40	0.8%	24 (60%)	16 (40%)
d530	Toileting	29	0.6%	15 (52%)	14 (48%)
d610	Acquiring a place to live	17	0.3%	6 (35%)	11 (65%)

*Categories are ordered by frequency in the dataset. Sample count and relative distribution between training data (229 documents, 3,350 samples) and test data (56 documents, 1,315 samples) are given for each category. Descriptions given are the preferred name of each category in the ICF*.

### Automated ICF Coding

#### Development Experiments: Identifying the Best Word Embeddings

**Figure 4:**
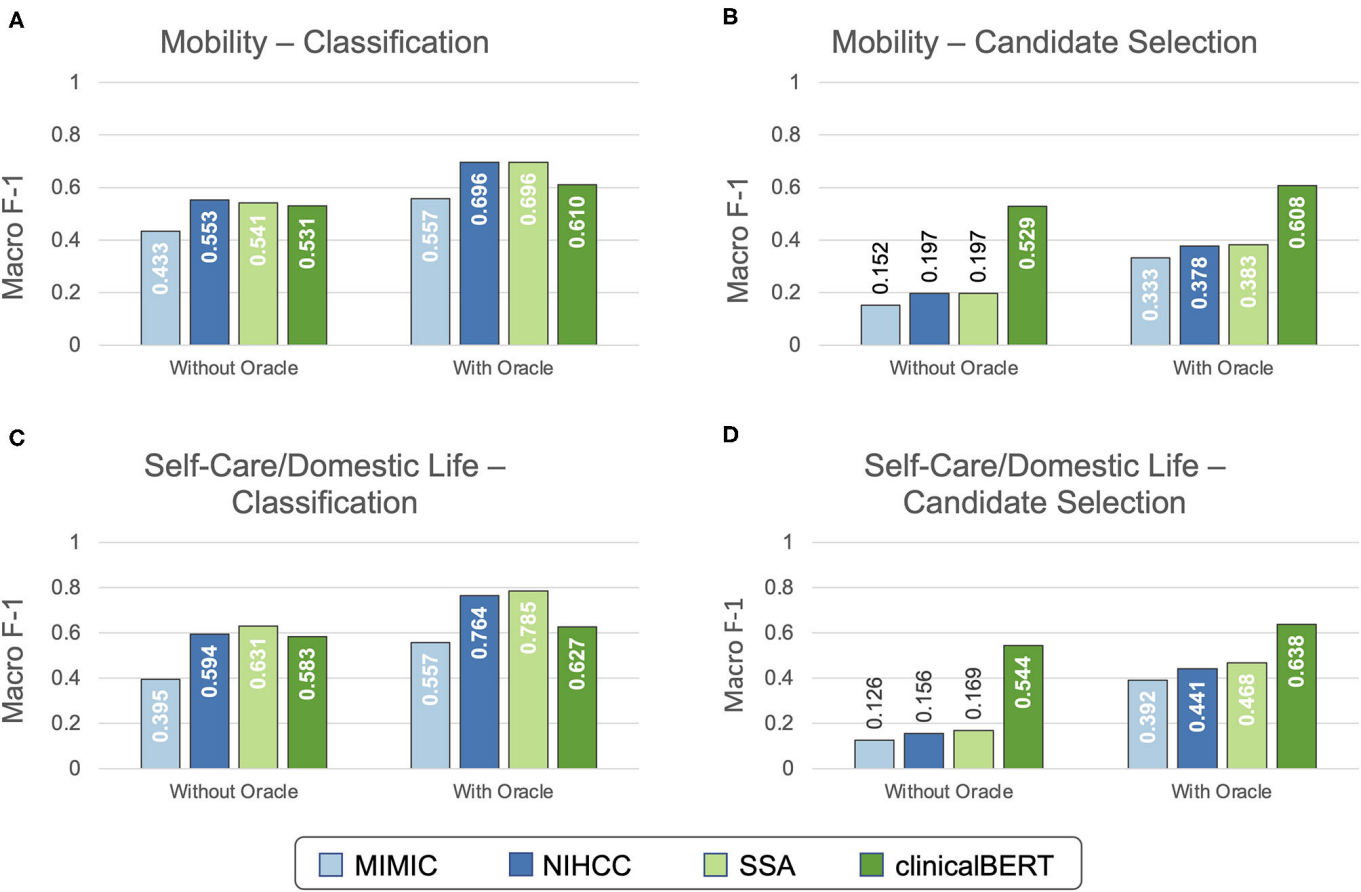
Development experiment results for selecting word embeddings. Development set performance (macro-averaged F-1 with 10-fold cross validation) is shown using each embedding strategy for both Mobility **(A,B)** and Self-Care/Domestic Life **(C,D)** data, using both classification **(A,C)** and candidate selection **(B,D)** approaches.

Figure 4 illustrates the results of development set experiments to identify the best word embedding features to use for coding Mobility and Self-Care/Domestic Life mentions. We evaluated MIMIC, NIHCC, SSA, and clinicalBERT embedding features for both classification and candidate selection approaches, with and without the Action oracle.

For the Mobility dataset, embeddings trained on the NIHCC and SSA corpora achieved highest development set performance both with the Action oracle (F-1 = 0.696 for both NIHCC and SSA) and without (NIHCC = 0.553, SSA = 0.541, difference not significant at *p*-value = 0.9, bootstrap resampling). NIHCC embeddings were statistically significantly better than the next best clinicalBERT features (F-1 of 0.553 vs. 0.531; *p-*value = 0.025) without the Action oracle, while SSA embeddings were not significantly different from clinicalBERT (F-1 of 0.541 vs. 0.531; *p*-value = 0.17). We, therefore, took NIHCC embeddings as the best-performing features for classification experiments on the Mobility test set.

For the Self-Care/Domestic Life dataset, SSA embeddings achieved highest development set performance both with the Action oracle (SSA F-1 = 0.785 vs. NIHCC F-1 = 0.764; *p*-value = 0.031) and without (SSA = 0.631, NIHCC = 0.594; *p*-value = 0.015). We, therefore, took SSA embeddings as the best-performing features for Self-Care/Domestic Life classification experiments.

Under the candidate selection approach, clinicalBERT features significantly (*p* ≪ 0.001) outperformed all other embeddings on both datasets. We used clinicalBERT embeddings as the best-performing features for test set candidate selection experiments.

#### Main Experiments

**Figure 5:**
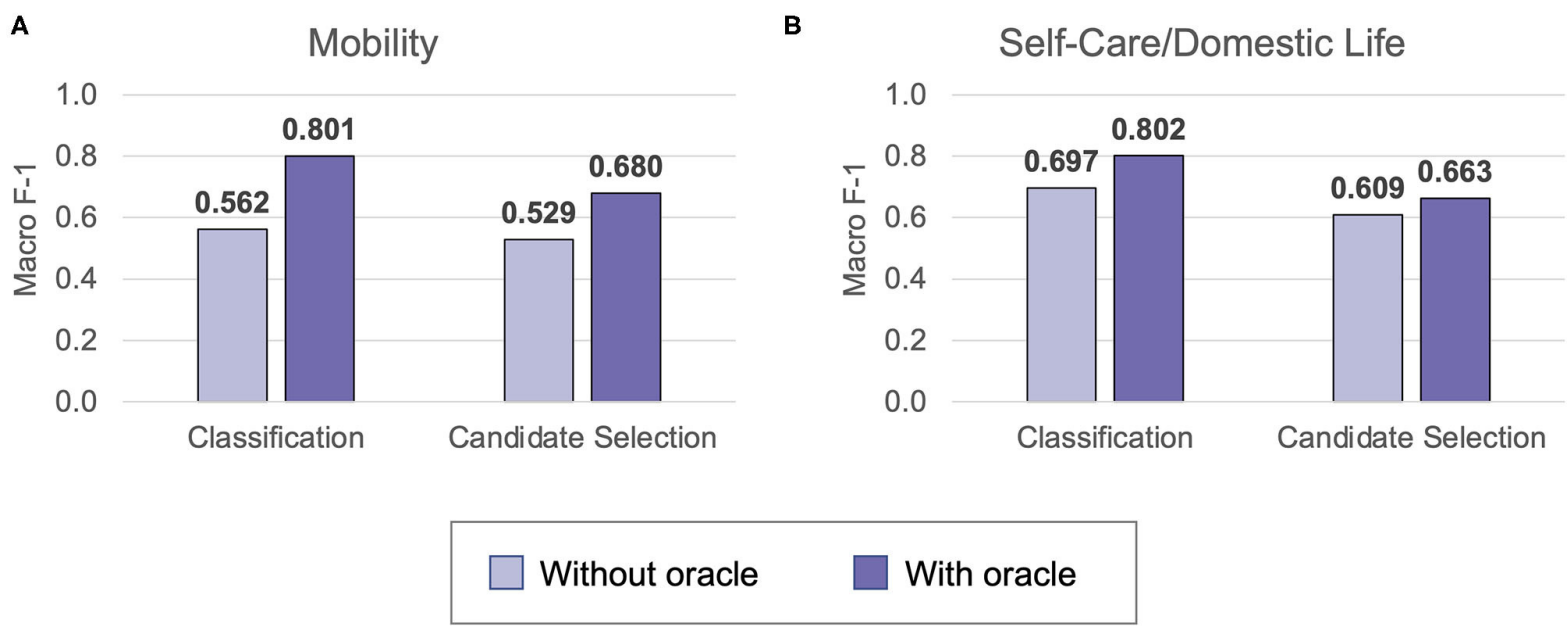
The test set performance on automated ICF coding in Mobility **(A)** and Self-Care/Domestic Life **(B)** test sets. Performance is reported for the best classification (Mobility: NIHCC embeddings; Self-Care/Domestic Life: SSA embeddings) and candidate selection (both datasets: clinicalBERT embeddings) models.

Figure 5 shows the overall performance of classification and candidate selection experiments on the Mobility and Self-Care/Domestic Life test sets. Classification models consistently outperformed candidate selection (*p* = 0.041 for Mobility without Action oracle; *p* ≪ *0.0*01 for Mobility with Action oracle and both settings of Self-Care/Domestic Life). This is consistent with our prior findings of comparable or slightly lower performance for our candidate selection model on Mobility data from physical therapy encounters (16). The Action oracle significantly (*p* ≪*0.0*01) improved performance in all cases, clearly demonstrating the value of building NLP systems to extract the Action components of activity mentions.

**Figure 6:**
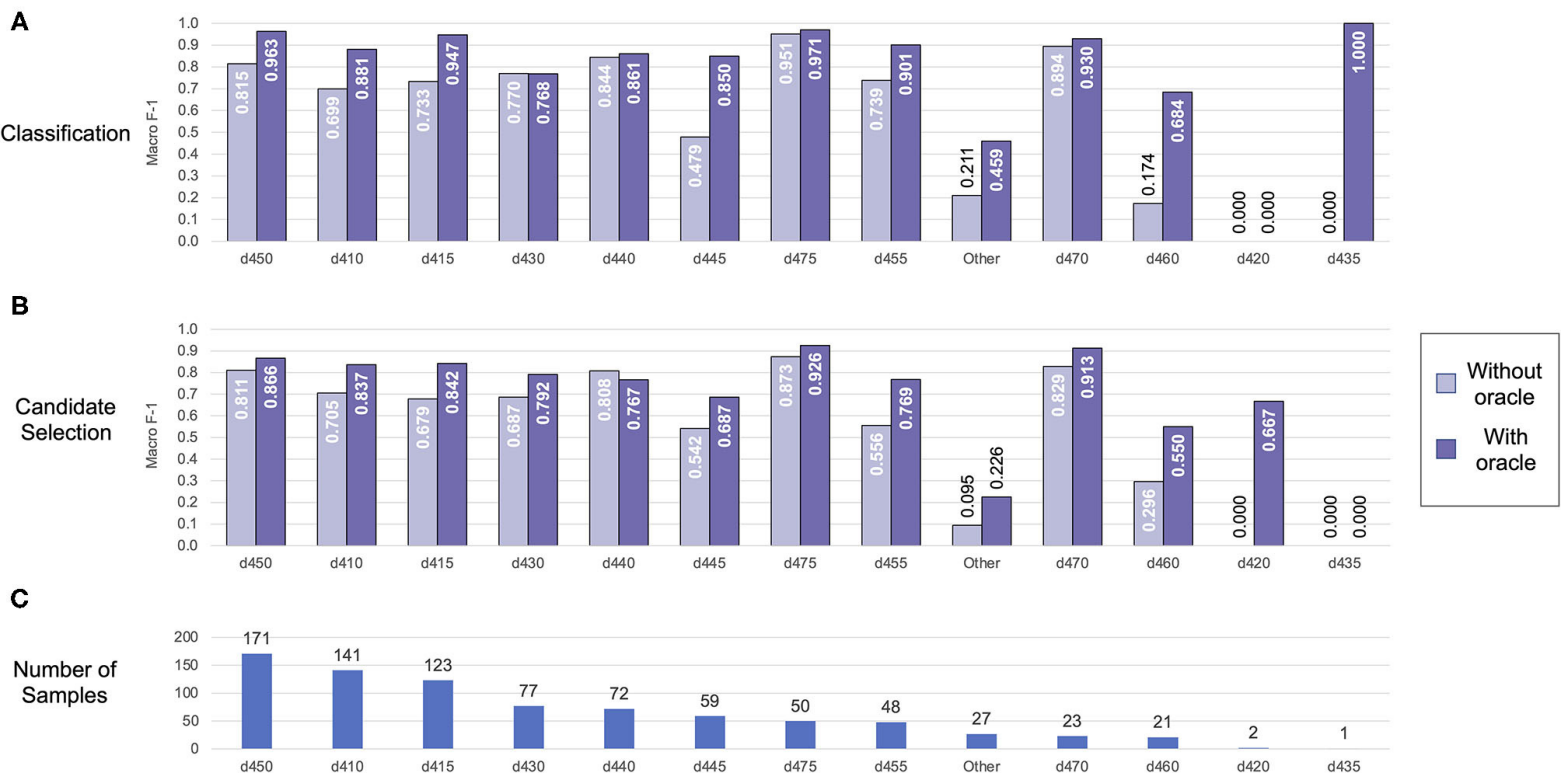
Automated coding performance for each distinct category in the Mobility dataset. Classification results are shown in **(A)**, and candidate selection results in **(B)**. Categories are ordered by descending frequency [illustrated in **(C)**].

**Figure 7:**
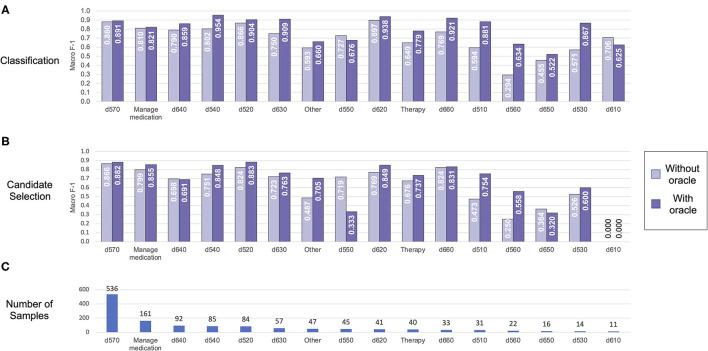
Automated coding performance for each distinct category in the Self-Care/Domestic Life dataset. Classification results are shown in **(A)**, and candidate selection results in **(B)**. Categories are ordered by descending frequency [illustrated in **(C)**].

We further analyzed performance on each individual label in the Mobility dataset (shown in Figure 6) and the Self-Care/Domestic Life dataset (shown in Figure 7). Performance generally trended with the frequency of the label—i.e., both classification and candidate selection performance was best for the most frequent categories and gradually degrades for less frequent categories. We did not observe any categories where our classification or candidate selection models showed a clear advantage; rather, our classification models tended slightly higher than candidate selection on almost all categories. Exposing the position of an Action component within an activity mention to the model (i.e., using the Action oracle) improved performance on almost all categories, with most of the largest gains on rare categories; e.g., an F-1 gain of 0.25 (candidate selection) and 0.5 (classification) on d460 (21 samples) in Mobility data, and an F-1 gain of 0.3 (candidate selection) and 0.33 (classification) on d560 (22 samples) in Self-Care/Domestic Life data.

## Discussion

We have shown that rich and diverse information on Mobility, Self-Care, and Domestic Life is recorded in free text health records collected from health providers by SSA for disability benefits adjudication. We presented NLP systems to map this information to specific ICF categories using two paradigms: classification (comparing each sample to other, previously seen samples) and candidate selection (comparing a sample to ICF categories directly). Our experiments demonstrated that these systems show promising performance for enabling automated analysis of medical evidence through the lens of the ICF.

Our study also revealed limitations of the ICF as a practical tool for analyzing medical documentation. We discuss key insights from our annotation process in the following section and highlight the particularly complex case of ICF category d570 *Looking after one's health*. We further identify particular successes and challenges arising from our NLP experiments and discuss implications of NLP tools for functional status, aligned with the ICF or with another conceptual framework, in both the SSA use case of disability adjudication and broader applications in clinical care and research.

### Practical Limitations of the ICF for Mobility, Self-Care, and Domestic Life Information

Coding functional status information according to a standardized framework such as the ICF allowed us to identify what kinds of functioning are discussed in health records and to organize information on patient functioning for retrieval and analysis. The ICF, as the internationally accepted classification of human functioning, is an important touchstone for this work, and it allowed us to capture a broad set of information about functional activity in free text health records. However, some activity mentions we observed in practice did not align with the categories presented in the ICF, such as “managing stairs,” “doing household tasks,” and “cleaning.” At the same time, other categories had significant overlap with one another in the expert annotation process, such as d450 *Walking*, d455 *Moving around*, and d460 *Moving around in different locations*. Category d465 *Moving around using equipment* was excluded entirely from annotation, as our information model represented assistive equipment (Assistance component) separately from the action being performed (Action component); this category, therefore, reduced to d455 *Moving around*. Some activity descriptors were highly context dependent for selecting the appropriate ICF category; for example, we annotated “drinking” as d560 *Drinking* for the generic action of drinking but as d570 *Looking after one's health* when used to refer specifically to drinking alcohol (e.g., “He drinks two shots of whiskey a day”). Thus, while the ICF is clear and comprehensive for coding many Mobility, Self-Care, and Domestic Life activities, its use is often more theoretical than practical when applied to actual clinical reporting.

#### ICF Category d570 Is Overly Broad

The limitations of the ICF in practice were particularly clear for the Self-Care category d570 *Looking after one's health*. We found this category to be significantly overrepresented in our data (accounting for 43.6% of all observed Self-Care/Domestic Life actions) and extremely broad in practice. Category d570 was treated as referring to preventative measures (e.g., exercising, taking prescribed medications, etc.) a person does to, or for, themselves or will/plans to do in the future. We excluded from consideration interventions performed or planned by healthcare providers, the goals providers set for themselves, and descriptions of specific therapy sessions that were not directly related to Self-Care. With this operational definition, we coded d570 for information as diverse as:

- She exercises four to five times a week.
- Stretching, breathing techniques
- He drinks two shots of whiskey a day.
- She has had two suicide attempts in the past.
- He smokes a pack of cigarettes a day.
- Takes over the counter supplements
- He is compliant with treatment but remains symptomatic.
- I haven't gone to counseling, but I talk to my friend who is a preacher.
- He consumed caffeine one to two times a week.

Notably, we found category d570 in practice to include several social determinants of health, such as drug and alcohol use (also including misuse and abuse) and smoking status. In addition to the breadth of information, several activity mentions we coded with d570 required some level of inference on the part of the reader to understand the functioning described. For example, we annotated “I talk to my friend who is a preacher” in the example above as d570, because, in the context of referring to counseling, this can be understood as the patient establishing a connection and/or reaching for help to look after themselves. References to suicide attempts were also coded as d570 because of the detriment to the physical and mental health of the patient.

From a practical standpoint in the annotation process, activity mentions coded with d570 presented two further complications. While stated (or implied) reasons for a patient taking care of themselves or not were not generally included in annotating activity mentions, in some cases, they provided context to clarify whether an action was related to taking care of oneself or not. For example, in “her tendency to take a double shift, knowing that there will be a detrimental impact on her comfort and health status,” the phrase “take a double shift” alone is not sufficient to determine a category of d570; including its effect on the health of the patient provides the necessary context to clarify that this is related to taking care of oneself. In addition, d570 was the only category where negation needed to be captured as part of the Action component when it pertained to suicide or other self-harm, recreational drug, and/or alcohol use, or medication non-compliance.

In summary, we found that the ICF is not necessarily in line with the types of information providers record about Self-Care, and that category d570 was too broad to effectively represent the diversity of Self-Care activities described in the data.

#### Distinguishing Patient Engagement in “Therapy” and “Manage Medications” From Other Uses of d570

We took the step in this study of specifically distinguishing patient engagement in *Therapy* (non-pharmacological) and *Manage medication* as distinct Self-Care categories, separate from the broader d570 category. We found that clinical notes frequently provided detailed information on how patients were or were not engaging actively in specific therapeutic interventions and determined that separate categories would provide a more organized view of the self-care activities of the patient as a whole. We distinguished between adherence to regimens for managing medications, which are therapies that a licensed provider needs to approve (in contrast to over-the-counter products, such as multivitamins or alternative medicines, which we classified as d570), and participation in non-pharmacological therapies, such as addiction treatment programs, physical therapy, occupational therapy, cognitive behavior modification therapy, psychological therapy and/or counseling, and anger management. To provide concrete examples of these distinctions and further illustrate the complex scope of category d570, Table 5 [drawn from our annotation guideline (32)] presents a selection of samples for each label, together with notes on why the information was or was not annotated as presented.

**Table 5:** Examples for the related labels of ICF category d570, Manage Medication, and Therapy.

**Category**	**Examples**	**Notes**
d570	Her sleep varies and she never feels rested	Not annotated; these fall within the Body Functions domain of the ICF.
	She has had a previous suicide attempt	Suicidal actions are annotated as indicating risks to health.
	He drinks a six-pack of beer a day	Reference to alcohol consumption.
	Patient was well-nourished	Indicates the person is taking care of themselves.
	Her tendency to take a double shift knowing that there will be a detrimental effect on her comfort and health status	Significant context is needed to clarify the impact on self-care.
Manage medication	He is currently prescribed medication by his neurologist to slow down the progression of his symptoms	Not annotated; does not state whether the person is actually taking the medications or not.
	$\text{Pt is currently }\underline{\text{on medication}}\text{:}$ Prazosin at bedtime…	Medications the patient is currently taking; the medications themselves are not annotated.
	She takes Tylenol	Reason for medication not needed; the specific medication is annotated to clarify what action is being performed.
Therapy	He has had no psychiatric care and no history of psychiatric hospitalization	Not annotated; reference to therapeutic care the patient has not used.
	$\text{She }\underline{\text{had occupational therapy}}$ for a custom splint	Therapy for a particular purpose related to health.
	$\text{He was }\underline{\text{seeing a counselor}}$ for his drug addiction	Counseling for a particular purpose related to health.

*Brief notes are provided for each example as to why it was or was not annotated as shown. Activity mentions are indicated using yellow highlights and Actions are indicated using underlines*.

#### Overlap Between d570 and Other Domains of the ICF

The interactions between health conditions, body functions and structures, activities and participation, and contextual factors are at the heart of the biopsychosocial model of the ICF of human function. However, we found that, particularly for category d570, both its definition and our observations of it in practice overlapped significantly with other domains of the ICF, creating an additional challenge for aligning clinical observations to the ICF model. Terms used in the definition of d570, such as “ensuring,” “appropriate level,” “avoiding harm,” and “being aware of the need,” are more aligned with the b1 *Mental functions* heading in the Body Functions domain. At the same time, several examples we annotated as d570 included elements more in the domain of Personal Factors—these included references to work preferences, physical activity levels, etc. As the ICF does not currently classify Personal Factors, these elements cannot be classified separately from the activity of d570. However, alternative models can also inform approaches to representing these relationships in practice; for example, the Institute of Medicine's 1997 model (33) separates personal factors into biologic factors (less modifiable) and lifestyle and behavior factors (more modifiable) and represents them as transitional factors in the enabling-disabling pathway. This perspective provides a framework for viewing the activity of *Looking after one's health* as an outwardly observable act affected by internal processes, such as personal health behaviors and choices. Modeling these relationships thus represents an important area of further inquiry both in refining the ICF model and in developing information technologies to align clinical observations with it.

#### Implications for Updating the ICF

Our findings suggest specific ways in which the ICF could be updated to decrease overlap between codes and better align with practical clinical reporting needs. Specific recommendations supported by our analysis include: (1) Remove the term “walking” from the definition of d460 *Moving around in different locations* to reduce overlap with d450 *Walking*. (2) Explicitly distinguish between the general action of drinking liquids, represented by category d560 *Drinking*, and the specific case of drinking alcohol (which providers often refer to simply using “drinking” or “drinks,” e.g., “his drinking habit” or “two drinks nightly”), which overlaps with d570 *Looking after one's health*. (3) Replace the broad category d570 *Looking after one's health* with multiple, more specific categories that reflect particular behavioral patterns, such as physical or cognitive exercises, substance use (ordered or disordered), or treatment compliance.

### NLP Is a Promising Technology for Analyzing FSI in Clinical Free Text

Our experiments demonstrate that NLP technologies can help to organize FSI in free text portions of the medical record, making this information easier to find and use in decision-making processes. Our findings identify particular opportunities for future work on refining and expanding these technologies, and we further discuss the potential implications of these technologies in managing SSA disability programs, as well as individual patient care.

#### Successes and Challenges in Automated ICF Coding With NLP

The natural language processing systems developed in this work achieved high performance for the majority of Mobility and Self-Care/Domestic Life ICF categories. The Action oracle was the single largest factor in system performance—F-1 on Mobility codes increased by 0.22, on average, for classification and 0.15, on average, for candidate selection; increases for Self-Care/Domestic Life were smaller but still considerable at 0.11 average for classification and 0.05 average for candidate selection. The first step in further refining NLP methods for analyzing FSI must, therefore, be to include identification of Action components in the process of extracting activity mentions from text.

On a per-category basis, the best NLP models achieved high performance for most ICF categories. In Mobility, we achieved over 0.9 F-1 for five high-impact categories: d450 *Walking*, d415 *Maintaining a body position*, d475 *Driving*, d455 *Moving around*, and d470 *Using transportation* (d435 *Moving objects with lower extremities* is not included in this list as only one sample was present in the test set, limiting the reliability of performance evaluations for this category). In Self-Care/Domestic Life, we exceeded 0.9 F-1 for five common categories: d540 *Dressing*, d520 *Caring for body parts*, d630 *Preparing meals*, d620 *Acquisition of goods and services*, and d660 *Assisting others*. System performance was not strongly correlated with the frequency of the ICF categories, indicating that, in most cases, there is a clear separation between categories. However, many of the errors made by all systems were mispredictions of the most frequent labels (d450 *Walking* for Mobility, d570 *Looking after one's health* for Self-Care/Domestic Life); frequency effects are thus still an important issue to address in further refinement of NLP models for ICF coding.

Per-category performance was more consistent for Self-Care/Domestic Life than for Mobility, despite the higher skew of the Self-Care/Domestic Life category distribution; this may reflect greater issues of category overlap in the Mobility domain. In both Mobility and Self-Care/Domestic Life data, the *Other* category was a consistent challenge, reflecting its nature as a catch-all category for samples that could not be mapped cleanly to single categories in the ICF.

#### Potential Applications in the SSA Disability Adjudication Process

The process of adjudicating applications to the SSA for federal disability benefits was one of the motivating use cases for this study. The adjudication process includes the collection and review of highly heterogeneous medical evidence, frequently collected as free text or semi-structured documents, to identify whether a person meets the necessary criteria for determining disability. This is a sequential process, which involves identifying information related to functioning at multiple steps. Claimants may be allowed based on meeting specified medical criteria organized into different body systems (34), where musculoskeletal criteria refer to several aspects of Mobility, criteria for mental disorders involve multiple areas of daily functioning, and criteria for multiple body systems refer to adherence to treatment. Claimants will also often report on daily activities and routines to provide details of functional abilities and limitations relevant to the workplace. Functional assessment is also a regular part of the adjudication process to determine whether a claimant is able to work, including through Residual Functional Capacity assessments, which include physical assessments highly dependent on Mobility. Thus, NLP-based tools to extract information related to functioning and organize it according to a standardized framework, such as the ICF, could be of use at multiple points in the disability adjudication process (35).

#### Broader Implications of ICF Coding With NLP

Natural language processing systems like the ones developed in this study have significant potential for helping to advance both clinical research and patient care. Identifying and organizing the rich information on individual function currently locked away in the medical free text can unlock valuable details to enrich the understanding of researchers of rehabilitation outcomes, and highlight salient details of experiences of patients in clinical decision-making. Prior research on automated and semi-automated ICD coding systems using NLP methods provides an instructive example of how these approaches can streamline medical coding processes (36–38). The growing integration of the ICF into clinical and research settings, from primary care (39) and EHR implementation (40) to pediatric research (41), presents similar opportunities to smooth the adoption and practical use of ICF categories with NLP-based coding systems. Vreeman and Richoz (42) describe potential benefits to both clinical care and research from integrating the ICF and other standardized vocabularies into EHRs, and Bettger et al. (43) highlight the role of EHR data in providing key insights to advance quality measures, research, and policy for rehabilitation. NLP technologies for ICF coding can serve as a valuable method to leverage the ICF as a lens to study the rich information collected in EHR notes.

In patient care, further development of NLP technologies can facilitate the decision-making process in several ways. Manabe et al. (44) developed an interactive system for selecting ICF categories in the EHR for mental health care; combining such an approach with NLP-based analysis could enable context-sensitive ICF coding during clinical note entry, improving the depth of information entered and its alignment with the ICF. At a new patient visit, NLP analysis of previously entered notes could also be used to highlight past limitations the patient experienced and inform patient-provider communication. Beyond the clinical setting, the use of NLP technologies for social support programs (such as the SSA disability programs that motivated our study) can help to more rapidly identify and organize key information from an individual's history to inform benefits decisions. Developing and evaluating new NLP technologies targeting further use cases in clinical research and patient care is a key direction for future research with significant potential for impact.

### Limitations

The SSA documents used in this study were a mix of clinical records sourced from healthcare providers around the U.S. and specialty records for consultations commissioned by SSA, pertaining to a disability benefits claim. These documents are thus not representative of EHR notes in most health systems. In addition, the population, who is the subject of these documents, consists of claimants for federal disability benefits due to work-related disability; this population is not necessarily representative of persons receiving rehabilitation care (or other care involving functional assessment) more broadly. From a practical standpoint, many of the SSA documents used exhibited severe noise from the OCR conversion process from scanned images to text. In our experiments, model design hyperparameters were not explored, nor were alternative classification or candidate selection methods, potentially limiting the F-1 measures we were able to achieve.

## Conclusions

Valuable information about patient functioning is regularly recorded in the free text portions of the EHR. The expressivity of natural language allows for the documentation of rich details about the functional experience, from levels of functional limitations experienced in different contexts to the goals and priorities of the patient for their own functioning. While free text documentation is difficult to analyze with traditional methods, NLP technologies enable a powerful, semantically enriched analysis of functioning information without losing expressivity. We analyzed two datasets of clinical records pertaining to disability benefits claims submitted to the U.S. Social Security Administration, using the ICF to identify and organize documented information about Mobility, Self-Care, and Domestic Life functioning of claimants. We found a rich diversity of functional status information in SSA documents and developed NLP models to automatically code this information according to the ICF. Our models achieved strong performance across key types of Mobility, Self-Care, and Domestic Life activities, demonstrating promise for automatically organizing functional status information within the ICF framework for easier analysis and review. We identified several practical limitations of the ICF for coding clinical reports, particularly the overly broad formulation of the Self-Care category d570 *Looking after one's health*. The results of this study and the NLP technologies assessed have significant implications for deepening the analysis of free text EHR data through an ICF lens and will contribute to ongoing efforts to learn more from the EHR in rehabilitation.

## Data Availability Statement

The datasets presented in this article are not readily available because they include identified medical information collected by the U.S. Social Security Administration for the purposes of adjudicating claims for disability benefits, and are not able to be shared. Requests for more information about the datasets should be directed to Julia Porcino, julia.porcino@nih.gov.

## Author Contributions

DN-G: conceptualization of the study, development of methodology, conducting experiments, data analysis, and the lead author of this manuscript. JC: development of methodology, data collection and annotation, and co-author of this manuscript. P-SH: development of methodology, data collection and annotation, and co-author of this manuscript. MS: development of methodology, data collection, and annotation. RJ: development of methodology, data collection and annotation, and statistical analysis. JP: project administration, development of methodology, data collection, and co-author of this manuscript. LC: acquisition of funding and project administration. All the authors contributed to this article and approved the submitted version.

## Funding

This research was supported by the Intramural Research Program of the National Institutes of Health and the U.S. Social Security Administration.

## Conflict of Interest

The authors declare that the research was conducted in the absence of any commercial or financial relationships that could be construed as a potential conflict of interest.

## Publisher's Note

All claims expressed in this article are solely those of the authors and do not necessarily represent those of their affiliated organizations, or those of the publisher, the editors and the reviewers. Any product that may be evaluated in this article, or claim that may be made by its manufacturer, is not guaranteed or endorsed by the publisher.

## Abbreviations

HER: electronic health record
FSI: functional status information
IAA: inter-annotator agreement
ICF: International Classification of Functioning, Disability, and Health
NLP: natural language processing
SSA: U.S. Social Security Administration.

## References

[1] World Health Organization. International Classification of Functioning, Disability and Health: ICF. Geneva: World Health Organization (2001).

[2] Gustavsen M Mengshoel AM. Clinical physiotherapy documentation in stroke rehabilitation: an ICIDH-2 beta-2 based analysis. Disabil Rehabil. (2003) 25:1089–96. 10.1080/0963828031000148629 12944148

[3] Bogardus ST Towle V Williams CS Desai MM Inouye S. What does the medical record reveal about functional status? J Gen Intern Med. (2001) 16:728–36. 10.1111/j.1525-1497.2001.00625.x 11722685 PMC1495285

[4] Nicosia FM Spar MJ Steinman MA Lee SJ Brown RT. Making function part of the conversation: clinician perspectives on measuring functional status in primary care. J Am Geriatr Soc. (2019) 67:493–502. 10.1111/jgs.15677 30506667 PMC6402957

[5] Rosenbloom ST Denny JC Xu H Lorenzi N Stead WW Johnson KB. Data from clinical notes: a perspective on the tension between structure and flexible documentation. J Am Med Inform Assoc. (2011) 18:181–6. 10.1136/jamia.2010.007237 21233086 PMC3116264

[6] Newman-Griffis D Porcino J Zirikly A Thieu T Camacho Maldonado J Ho P-S. Broadening horizons: the case for capturing function and the role of health informatics in its use. BMC Public Health. (2019) 19:1288. 10.1186/s12889-019-7630-3 31615472 PMC6794808

[7] Kimia AA Savova G Landschaft A Harper MB. An introduction to natural language processing: how you can get more from those electronic notes you are generating. Pediatr Emerg Care. (2015) 31:536–41. 10.1097/PEC.0000000000000484 26148107

[8] Kreimeyer K Foster M Pandey A Arya N Halford G Jones SF. Natural language processing systems for capturing and standardizing unstructured clinical information: a systematic review. J Biomed Inform. (2017) 73:14–29. 10.1016/j.jbi.2017.07.012 28729030 PMC6864736

[9] Kukafka R Bales ME Burkhardt A Friedman C. Human and automated coding of rehabilitation discharge summaries according to the international classification of functioning, disability, and health. J Am Med Informatics Assoc. (2006) 13:508–15. 10.1197/jamia.M2107 16799117 PMC1561799

[10] Agaronnik N Lindvall C El-Jawahri A He W Iezzoni L. Use of natural language processing to assess frequency of functional status documentation for patients newly diagnosed with colorectal cancer. JAMA Oncol. (2020) 6:1628–30. 10.1001/jamaoncol.2020.2708 32880603 PMC7489406

[11] Agaronnik ND Lindvall C El-Jawahri A He W Iezzoni LI. Challenges of developing a natural language processing method with electronic health records to identify persons with chronic mobility disability. Arch Phys Med Rehabil. (2020) 101:1739–46. 10.1016/j.apmr.2020.04.024 32446905 PMC7529728

[12] Chen T Dredze M Weiner JP Hernandez L Kimura J Kharrazi H. Extraction of geriatric syndromes from electronic health record clinical notes: assessment of statistical natural language processing methods. JMIR Med Inf. (2019) 7:e13039. 10.2196/13039 30862607 PMC6454337

[13] Newman-Griffis D Zirikly A. Embedding transfer for low-resource medical named entity recognition: a case study on patient mobility. In: Proceedings of the BioNLP 2018 workshop. Melbourne, Australia: Association for Computational Linguistics. (2018). p. 1–11. 10.18653/v1/W18-2301

[14] Newman-Griffis D Fosler-Lussier E. HARE: a flexible highlighting annotator for ranking and exploration. In: Proceedings of the 2019 Conference on Empirical Methods in Natural Language Processing and the 9th International Joint Conference on Natural Language Processing (EMNLP-IJCNLP): System Demonstrations. Hong Kong, China: Association for Computational Linguistics. (2019). p. 85–90. 10.18653/v1/D19-3015 PMC7731636 33313604

[15] Thieu T Maldonado JC Ho P-S Ding M Marr A Brandt D. A comprehensive study of mobility functioning information in clinical notes: entity hierarchy, corpus annotation, and sequence labeling. Int J Med Inform. (2021) 147:104351. 10.1016/j.ijmedinf.2020.104351 33401169 PMC8104034

[16] Newman-Griffis D Fosler-Lussier E. Automated coding of under-studied medical concept domains: linking physical activity reports to the international classification of functioning, disability, and health. Front Digit Heal. (2021) 3:620828. 10.3389/fdgth.2021.620828 33791684 PMC8009547

[17] Kohler F Connolly C Sakaria A Stendara K Buhagiar M Mojaddidi M. Can the ICF be used as a rehabilitation outcome measure? A study looking at the inter- and intra-rater reliability of ICF categories derived from an ADL assessment tool. J Rehabil Med. (2013) 45:881–7. 10.2340/16501977-1194 23979649

[18] den Ouden MEM Schuurmans MJ Mueller-Schotte S Brand JS van der Schouw YT. Domains contributing to disability in activities of daily living. J Am Med Dir Assoc. (2013) 14:18–24. 10.1016/j.jamda.2012.08.014 23043731

[19] Linacre JM Heinemann AW Wright BD Granger C V Hamilton BB. The structure and stability of the functional independence measure. Arch Phys Med Rehabil. (1994) 75:127–32. 10.1016/0003-9993(94)90384-0 8311667

[20] Hripcsak G Rothschild AS. Agreement, the F-measure, and reliability in information retrieval. J Am Med Informatics Assoc. (2005) 12:296. 10.1197/jamia.M1733 15684123 PMC1090460

[21] Grouin C Rosset S Zweigenbaum P Fort K Galibert O Quintard L. Proposal for an extension of traditional named entities: from guidelines to evaluation, an overview. In: Proceedings of the 5th Linguistic Annotation Workshop. Portland, Oregon, USA: Association for Computational Linguistics. (2011). p. 92–100.

[22] Turney PD Pantel P. From frequency to meaning: vector space models of semantics. J Artif Intell Res. (2010) 37:141–88. 10.1613/jair.2934

[23] Bojanowski P Grave E Joulin A Mikolov T. Enriching word vectors with subword information. Trans ACL. (2017) 5:135–46. 10.1162/tacl_a_00051

[24] Devlin J Chang M-W Lee K Toutanova K. BERT: pre-training of deep bidirectional transformers for language understanding. In: Proceedings of the 2019 Conference of the North American Chapter of the Association for Computational Linguistics: Human Language Technologies, Volume 1 (Long and Short Papers). Minneapolis, Minnesota: Association for Computational Linguistics. (2019). p. 4171–86.

[25] Honnibal M Montani I. spaCy 2: natural language understanding with Bloom embeddings, convolutional neural networks and incremental parsing. To Appear. (2017) 7:411–20. 10.3233/978-1-60750-588-4-1080

[26] Johnson AEW Pollard TJ Shen L Lehman L-WH Feng M Ghassemi M. MIMIC-III, a freely accessible critical care database. Sci Data. (2016) 3:160035. 10.1038/sdata.2016.35 27219127 PMC4878278

[27] Alsentzer E Murphy J Boag W Weng W-H Jindi D Naumann T. Publicly available clinical BERT embeddings. In: Proceedings of the 2nd Clinical Natural Language Processing Workshop. Minneapolis, Minnesota, USA: Association for Computational Linguistics. (2019). p. 72–8. 10.18653/v1/W19-1909

[28] Noble WS. What is a support vector machine? Nat Biotechnol. (2006) 24:1565–7. 10.1038/nbt1206-1565 17160063

[29] Berg-Kirkpatrick T Burkett D Klein D. An empirical investigation of statistical significance in NLP. In: Proceedings of the 2012 Joint Conference on Empirical Methods in Natural Language Processing and Computational Natural Language Learning. Association for Computational Linguistics. (2012). p. 995–1005. Available online at: http://aclweb.org/anthology/D12-1091

[30] Dror R Baumer G Shlomov S Reichart R. The hitchhiker's guide to testing statistical significance in natural language processing. In: Proceedings of the 56th Annual Meeting of the Association for Computational Linguistics (Volume 1: Long Papers). Association for Computational Linguistics. (2018). p. 1383–92. Available online at: http://aclweb.org/anthology/P18-1128 10.18653/v1/P18-1128

[31] Soberg HL Sandvik L Ostensjo S. Reliability and applicability of the ICF in coding problems, resources and goals of persons with multiple injuries. Disabil Rehabil. (2008) 30:98–106. 10.1080/09638280701216862 18189163

[32] Epidemiology & Biostatistics Section, Rehabilitation Medicine Department NI of HCC. Formulating Functional Terminology from the International Classification of Functioning, Disability and Health: Annotation Guideline for Self-Care and Domestic Life Domains. (2020).

[33] Institute of Medicine. Enabling America: Assessing the Role of Rehabilitation Science and Engineering. Pope AM Brandt EN editors. Washington, DC: National Academy Press (1997). 25121279

[34] US Social Security Administration. Disability Evaluation Under Social Security. 64-039. (2008). Available online at: https://www.ssa.gov/disability/professionals/bluebook/general-info.htm. (accessed August 29, 2019).

[35] Desmet B Porcino J Zirikly A Newman-Griffis D Divita G Rasch E. Development of natural language processing tools to support determination of federal disability benefits in the U.S. In: Proceedings of the 1st Workshop on Language Technologies for Government and Public Administration (LT4Gov). Marseille, France: European Language Resources Association. (2020). p. 1–6. Available online at: https://www.aclweb.org/anthology/2020.lt4gov-1.1

[36] Imai T Kajino M Sato M Ohe K. Development of structured ICD-10 and its application to computer-assisted ICD coding. Stud Health Technol Inform. (2010) 160:1080–4. 20841850

[37] Harteloh P. The implementation of an automated coding system for cause-of-death statistics. Inform Health Soc Care. (2020) 45:1–14. 10.1080/17538157.2018.1496092 30125131

[38] Zhou L Cheng C Ou D Huang H. Construction of a semi-automatic ICD-10 coding system. BMC Med Inform Decis Mak. (2020) 20:67. 10.1186/s12911-020-1085-4 32293423 PMC7157985

[39] Postma SAE van Boven K Ten Napel H Gerritsen DL Assendelft WJJ Schers H. The development of an ICF-based questionnaire for patients with chronic conditions in primary care. J Clin Epidemiol. (2018) 103:92–100. 10.1016/j.jclinepi.2018.07.005 30009942

[40] Maritz R Aronsky D Prodinger B. The international classification of functioning, disability and health (ICF) in electronic health records: a systematic literature review. Appl Clin Inform. (2017) 8:964–80. 10.4338/ACI-2017050078 28933506 PMC6220695

[41] Schiariti V Longo E Shoshmin A Kozhushko L Besstrashnova Y Król M. Implementation of the international classification of functioning, disability, and health (ICF) core sets for children and youth with cerebral palsy: global initiatives promoting optimal functioning. Int J Environ Res Public Health. (2018) 15:1899. 10.3390/ijerph15091899 30200412 PMC6163506

[42] Vreeman DJ Richoz C. Possibilities and implications of using the ICF and other vocabulary standards in electronic health records. Physiother Res Int. (2015) 20:210–9. 10.1002/pri.1559 23897840 PMC3907616

[43] Bettger JP Nguyen VQC Thomas JG Guerrier T Yang Q Hirsch MA. Turning data into information: opportunities to advance rehabilitation quality, research, and policy. Arch Phys Med Rehabil. (2018) 99:1226–31. 10.1016/j.apmr.2017.12.029 29407515 PMC6571032

[44] Manabe S Miura Y Takemura T Ashida N Nakagawa R Mineno T. Development of ICF code selection tools for mental health care. Methods Inf Med. (2011) 50:150–7. 10.3414/ME10-01-0062 21170470

